# Association Between the Work Environment and Nurses’ Resilience in Selected Geriatric Facilities in Hanoi, Vietnam

**DOI:** 10.1155/jonm/1362411

**Published:** 2026-09-25

**Authors:** Thuy Thi Ngo, Lydia T. Manahan, Edreck D. Estioko, Huong Thi Thu Pham

**Affiliations:** ^1^ Faculty of Medicine and Pharmacy, Intracom University, Hung Yen, Vietnam; ^2^ College of Nursing, Trinity University of Asia, Quezon City, Philippines; ^3^ Faculty of Nursing, Phenikaa School of Medicine and Pharmacy, Phenikaa University, Hanoi, Vietnam, phenikaa-uni.edu

**Keywords:** geriatrics, nurse resilience, nursing leadership, nursing work environment, Vietnam

## Abstract

**Background:**

Global healthcare systems face increasing pressure from rapidly ageing populations and persistent nursing workforce shortages. These challenges are particularly pronounced in geriatric care, where nurses must meet complex clinical demands while sustaining long‐term, emotionally intensive care.

**Aim:**

This study aimed to examine the relationship between the nursing work environment and resilience among nurses working in geriatric facilities in Hanoi, Vietnam.

**Methods:**

A descriptive, cross‐sectional correlational design was employed. Data were collected from 222 nurses across six geriatric facilities in Hanoi. The Practice Environment Scale of the Nursing Work Index (PES‐NWI, short version) and a standardised resilience measurement tool were used. Pearson’s correlation coefficient was applied to assess associations between variables.

**Results:**

Nurses perceived their work environment as generally supportive, particularly regarding leadership and care quality, despite lower ratings for resource adequacy. A statistically significant moderate positive correlation was found between the overall work environment and nurse resilience (*r* = 0.534, *p* = 0.001). Subdomain analyses revealed a nuanced pattern: while a strong focus on nursing care was positively associated with stress management capacities, it was inversely related to aspects reflecting personal balance, suggesting potential tensions between professional adaptability and personal boundary maintenance.

**Conclusion:**

A supportive work environment is fundamentally associated with higher resilience among geriatric nurses. However, resilience in this context appears multidimensional, whereby professional coping strengths may coexist with challenges in maintaining personal balance. Organisational support is therefore essential not only for enhancing resilience but also for sustaining the long‐term well‐being of the geriatric nursing workforce.

**Implications for Nursing Management:**

Nursing managers should prioritise culturally responsive leadership and participatory decision‐making while addressing structural factors such as staffing and resource allocation. Organisational interventions that balance professional demands with personal sustainability are critical to fostering resilient geriatric nursing practice.

## 1. Introduction

### 1.1. Global and Managerial Context

Ageing of the population is one of the most drastic demographic changes of the twenty‐first century, with far‐reaching consequences to the healthcare systems of the world. Many elderly adults have a combination of chronic health problems, functional and psychosocial requirements that necessitate a complex and continuous nursing support [1]. The increasing demand for geriatric care is expected to result in more nurses becoming overworked. Meanwhile, persistent shortages of nursing workforce, high turnover rates and rising rates of work‐related stress and burnout are becoming common in most countries. Those challenges are the most acute in geriatric environments where nurses are prone to excessive workloads, emotional labour and their limited organisational resources.

From a nursing management perspective, geriatric care sustainability lies not only on the number of staff but also on the quality of the nursing working environment [2]. The organisational cultures include organisational structures, leadership styles, work relationships and availability of resources that all contribute to the daily experiences of the nurses [3, 4]. Experiments have repeatedly shown that poor working conditions lead to job discontent [5], poor performance, turnover intentions [6] and otherwise negative health, job involvement and satisfactory care [7]. The enhancement of the nursing work environment has become one of the strategic priorities of healthcare managers in need to enhance workforce stability and care outcomes [8].

In its framework, the concept of resilience has acquired more and more importance as a psychological and professional skill of nurses who work in high‐demand environments [9]. The notion of resilience can be described as the capacity to adapt in a positive way to adversity, continue functioning in the profession and be able to recuperate under the influence of stress [10]. Resiliency is especially significant to geriatric nurses as they are subject to complicated illness management, palliative care and emotional burnout [2, 11]. Resilience can no longer be regarded as a single characteristic of an individual; the organisational conditions, the support of leaders and the culture in a working place determine it [9]. The comprehension of the impact of the nursing work environment on resilience is paramount to effective nursing management and workforce planning.

### 1.2. Nursing Work Environment and Resilience

The nursing work environment has entered into the realm of intensive research as one of the influencers and predictors of nurse outcomes and quality of patient care. Among the common key dimensions of leadership and management support, nurse involvement in organisational decision‐making, sufficiency of staffing and resources and quality of interdisciplinary relationships are represented ([4], p. 842). Achievement of positive work environments has been attributed to supportive leadership, professional autonomy, teamwork and adequate material and human resources. On the other hand, a poor work environment is linked to heavy workloads, minimal managerial assistance, role conflicts and poor communication. These interconnected findings underscore how suboptimal workplace environments can fundamentally undermine nurses’ job satisfaction, organizational performance, and healthcare delivery.

The conceptualisation of resilience as a dynamic process has made it possible to consider individuals as able to cope with stressors and stay psychologically healthy and professionally competent. Resilience in nursing has been linked to low burnout, elevated levels of job satisfaction and the ability to provide quality care at any given moment [9, 11]. New studies indicate that organisational drivers are very important towards the development of nurse resilience. Positive team dynamics, pro‐professional development and supportive leadership practises have been identified as having a strong capacity to strengthen the adaptive capabilities of nurses [3]. On the other hand, the presence of resilience‐threatening environments that are chronically understaffed and lack effective leadership and recognition can expose nurses to emotional burnout and detachment.

Although there is an increased awareness about the interconnectedness between the workplace and resilience, a large portion of the available research is based on the acute care context of high‐income nations. The literature on geriatric care settings is underrepresented, especially in the low‐ and middle‐income countries [2]. There have been numerous studies that have centred on resilience interventions at the individual level, that is, mindfulness or coping skills training, but have not given enough consideration to organisational and managerial factors ([12], p. 929). This gap restricts the evidence‐based development of management strategies that are supposed to help to build resilience by changing the work environment.

Resilience in the nursing environment is not a matter of burnout, job satisfaction and turnover, but the capacity to respond to adversity in a constructive way, emotional balance and functioning with stressors. Research, including Aqtam et al. [13], discovered a negative association between stress and resilience among nurses in the ICU, which implied that high stress levels at the workplace are associated with a detrimental effect on resilience. On the same note, Ayed et al. [14] found that work environment conditions in the ICU environment modulate the professional quality of life of nurses, including outcomes of resilience.

New data in high‐demand clinical facilities also lend credence to the connection between workplace stressors and nurse resilience. In a cross‐sectional study that included nurses in the intensive care unit, it was found that there was a moderate negative correlation between stress and resiliency, indicating organisational and environmental factors in the development of adaptive capacity [13]. Likewise, research that investigates the topic of professional quality of life has also shown that work environment variables, such as leadership, staffing satisfaction and team dynamics, have significant correlations with the outcomes of nurse well‐being, namely burnout, compassion satisfaction and secondary traumatic stress [14, 15]. Further studies have revealed that patient‐centred care and the quality of care overall have a positive correlation with supportive practice environments [16]. Also, the recent analysis of critical care facilities has shown that resilience is tightly connected with experiencing psychological distress, which justifies officials of supportive working patterns to help alleviate negative consequences in the nurses [17].

### 1.3. Geriatric Nursing and Leadership Challenges in Vietnam

Vietnam is experiencing a rapid demographic transition, with a steadily increasing ageing population that has intensified the demand for long‐term and specialised geriatric care. In response, geriatric healthcare services have expanded, particularly in major urban centres such as Hanoi and Ho Chi Minh City. Nevertheless, this growth has occurred within a healthcare system that continues to face significant constraints, including limited resources, workforce shortages and unequal access to specialised geriatric education [1]. Geriatric nursing remains an emerging field in Vietnam, and existing evidence indicates persistent gaps in formal geriatric education and training among healthcare providers, including nurses, which may limit their preparedness to address the complex and multidimensional needs of older adults [18]. These gaps are further reflected in preservice nursing education, where nursing students report limited geriatric knowledge and variable willingness to care for older people, suggesting structural challenges in the geriatric care training pipeline [19].

Nursing management and leadership within Vietnamese geriatric care facilities face challenges similar to those observed in other acute care settings, such as chronic understaffing, high nurse‐to‐patient ratios, limited opportunities for professional development and hierarchical organisational structures that may restrict nurses’ participation in decision‐making processes [3]. Evidence from Vietnam and comparable healthcare contexts indicates that geriatric nurses often experience lower professional recognition and fewer incentives than those working in acute or highly specialised settings, contributing to reduced job satisfaction, diminished morale and increased turnover intention [2, 5].

While hierarchical and collectivist cultural norms may support organisational stability, they can also constrain open communication and shared decision‐making when inadequately managed. Examining the interaction among cultural context, nursing work environment and nurse resilience is therefore essential to inform the development of culturally responsive management strategies that foster supportive work environments and sustain the geriatric nursing workforce in Vietnam. The six geriatric facilities used in this study are largely urban geriatric care environments in Hanoi and might not be as representative of the breadth of provision of geriatric care in rural or less‐resourced parts of Vietnam. It is necessary to mention that the six geriatric facilities that will be used in this study are all in Hanoi, which is an urban geriatric care setting. The results might not be representative of the situations in less‐resourced or rural regions of Vietnam.

### 1.4. Knowledge Gap and Justification

Even though international studies have detected the correlation between nursing work conditions and nurse‐related outcomes, evidence from Vietnam remains markedly limited, particularly in geriatric care settings [2]. Existing research in Vietnam has primarily focused on general job satisfaction, burnout or geriatric knowledge among healthcare providers, while resilience as an adaptive capacity in nursing practice has received comparatively little empirical attention [9, 18]. Moreover, the literature remains fragmented in examining how organisational and managerial factors shape resilience among nurses working with older adults.

This management‐oriented research gap—linking established nursing theories with empirical evidence on work environments and resilience—constrains the ability of nurse managers and policymakers to develop context‐specific organisational strategies that support a resilient geriatric nursing workforce [1]. Addressing this gap is therefore critical to informing leadership practices, workforce planning and policy formulation aimed at strengthening the sustainability and quality of geriatric care services in Vietnam. The study by the authors is the first‐ever research on Vietnamese geriatric facilities to investigate relations between the work environment domains of PES‐NWI and resilience domains of RAW among nurses. This study is the first to examine the correlation between PES‐NWI domains and RAW resilience domains in Vietnamese geriatric facilities.

### 1.5. Aim and Research Questions

This study was conducted to determine relationships between the nursing working environment and resilience among geriatric nurses working in a geriatric facility in Hanoi, Vietnam. The researchers aimed at evaluating the perception of the work environment by nurses, measuring nurse resilience and examining the associations between work environment dimensions and resilience outcomes. By meeting these goals, the research paper provides empirical results to reinforce the use of the nursing management strategies to build resilience by enhancing the organisational conditions.

## 2. Theoretical and Conceptual Framework

### 2.1. Neuman Systems Model

The Neuman systems model provides a comprehensive theoretical framework for understanding the dynamic interactions between individuals and their environments [20]. Within this model, individuals are conceptualised as open systems that continuously respond to internal and external stressors. These stressors are classified as intrapersonal, interpersonal and extrapersonal, with their effects mediated by the strength of the flexible line of defence and the lines of resistance. Nursing interventions are therefore directed towards reducing stressor exposure and strengthening protective resources to support system stability and recovery.

In the context of the present study, the nursing work environment can be conceptualised as a salient extrapersonal stressor shaping nurses’ professional functioning and psychological well‐being. Adverse organisational conditions, including inadequate leadership support, resource constraints and staffing shortages, have been shown to be associated with heightened occupational stress among geriatric nurses and may compromise their adaptive capacity [2]. Conversely, supportive leadership practices, positive professional relationships and adequate organisational resources function as protective factors that help buffer the impact of workplace stressors and support reconstitution following stress exposure [3]. The Neuman systems model, therefore, offers a theoretically robust lens for examining how organisational environments influence nurse resilience through mechanisms of stress exposure, protection and recovery.

### 2.2. Nursing Resilience Theory

Nursing resilience theory conceptualises the meaning of resilience in terms of a dynamic process that varies according to circumstances as opposed to being a trait‐based individual characteristic. As per this view, resilience occurs as a result of the interaction between personal resources and environmental resources [10]. Nurses acquire resilience based on experiences that contribute to better coping, meaning‐making and professional development, especially where these experiences are fostered by positive organisational conditions [9, 11].

Management‐wise, this theory has given special consideration to the importance of the management and organisational culture in enhancing resilience. Resilience can be enhanced through providing supportive supervision, promoting participation in decision‐making and acknowledging the contributions of nurses to build professional identity and self‐efficacy [3]. On the contrary, neglectful, overdemanding and undersupportive environments can wear down resilience over time. The application of the nursing resilience theory to the geriatric setting highlights the significance of organisational policies that transcend interventions dealing with individual levels and deal with systemic issues of the workplace [2].

### 2.3. Conceptual Model of the Study

This study’s conceptual model was developed by combining the Neuman systems model and nursing resilience theory. The Neuman systems model understands the person as an open system that is continually engaged in interacting with internal and external stressors. The setting of the nursing job is conceptualised in this study as an important extrapersonal organisational factor, which can either potentially increase the stress exposure or can act as a protective resource for the nurse in the geriatric care facility.

Flexible lines of defence and adaptive responses to occupational stress can be reinforced by supportive leadership, appropriate staffing and resources, involvement in decision‐making, collaboration among professionals and building a solid base for quality nursing care. On the other hand, the lack of resources, lack of involvement in management, lack of staffing and poor organisational support can heighten the exposure to stress and impact nurses’ capacity to achieve professional and personal balance.

The nursing resilience theory reinforces the concept that resilience is not a trait of the individual but a process that is influenced by personal attributes and the conditions of the workplace. Nurse resilience, as a result of this study, is therefore conceptualised as an adaptive response, determined by organisational and managerial factors, in geriatric care units.

**FIGURE 1:**
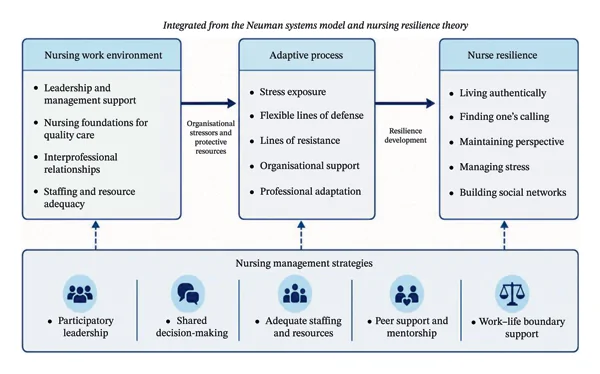
Strengthening work resilience framework.

The conceptual model suggests that three aspects of the work environment of nurses are associated with nurse resilience: leadership and management support, nursing foundations for quality of care and interprofessional working and staffing and resource adequacy. The work environment dimensions are hypothesised to relate to work dimensions of resilience, such as living authentically, finding one’s calling, maintaining perspective, managing stress and building social networks. As shown in Figure 1, the conceptual framework proposes that three dimensions of the nursing work environment—leadership and management support, nursing foundations for quality care and interprofessional relationships and staffing and resource adequacy—are associated with nurse resilience domains, including living authentically, finding one’s calling, maintaining perspective, managing stress and building social networks.

Therefore, positive relationships between the supportive nursing work environment and overall nurse resilience are expected. Resilience is multifaceted, however, and various aspects of the work environment may be associated with various aspects of resilience. For instance, good interprofessional working can be helpful in developing nurses’ capacity for self‐care and managing stress in the workplace, while poor staffing levels and overloading workload can undermine their capacity for maintaining perspectives, personal balance and boundaries around their work.

This conceptualisation informed the study by focusing the analysis on the independent organisational variable – the nursing work environment – and the dependent adaptive outcome – nurse resilience (see Table 1). The model also highlights the importance of not only acquiring individual coping strategies but also of implementing organisational interventions through nursing management to build resilience in geriatric nursing. Thus, enhanced leadership, resource management, interprofessional working and nurse involvement in decision‐making could help to strengthen and sustain a geriatric nursing workforce.

**TABLE 1:** Mapping of theoretical concepts to study variables.

Theoretical concept	Study variable	Operational meaning in this study
Extrapersonal stressors and protective factors from the Neuman systems model	Nursing work environment	Organisational conditions that may either increase workplace stress or support nurses’ adaptive functioning
Leadership and management support	PES‐NWI leadership and management dimension	Managerial support, participation in decision‐making, continuing education and quality improvement activities
Professional relationships and nursing foundations for quality care	PES‐NWI nursing foundations and interdisciplinary relationships dimension	Shared nursing philosophy, nurse competence, continuity of care and nurse–physician collaboration
Structural resources	PES‐NWI staffing and resource adequacy dimension	Availability of sufficient staff and qualified nurses to meet geriatric care demands
Adaptive response from nursing resilience theory	Nurse resilience	Nurses’ capacity to adapt positively to workplace demands and sustain professional functioning
Personal and professional resilience domains	RAW subscales	Living authentically, finding one’s calling, maintaining perspective, managing stress and building social networks

Abbreviations: PES‐NWI, Practice Environment Scale of the Nursing Work Index; RAW, Resilience at Work Scale.

## 3. Methods

### 3.1. Study Design

This study employed a descriptive correlational cross‐sectional design to examine the association between the nursing work environment and resilience among nurses working in geriatric care settings. This design is appropriate for assessing relationships between key variables at a single point in time without experimental manipulation of the study context, particularly in applied healthcare research [21].

Cross‐sectional correlational approaches have been widely used in nursing and healthcare management research to investigate organisational factors and workforce‐related outcomes, including leadership, empowerment and staff commitment [3]. Furthermore, such designs are considered suitable for exploratory research conducted in underresearched or emerging practice contexts, including geriatric nursing, where baseline empirical evidence remains limited [2].

### 3.2. Setting

The study was carried out in six geriatric healthcare centres in Hanoi, Vietnam. Hanoi was chosen because it is one of the most important cities in the country, and as a result of the population ageing, it is seeing a growing demand for geriatric and long‐term care services. The facilities involved took care of older adults with chronic diseases, functional impairments, rehabilitation needs and long‐term nursing care needs.

There were six facilities, consisting of public and semipublic geriatric care institutions. While not identical in bed size, staffing structure or resources, they were all facilities that directly provided nursing care to older adults, and had common staffing issues—high demands in care, low staffing levels and resource issues. They provided services including long‐term residential care, management of chronic disease, medicine administration, support for basic rehabilitation, assistive services and support with activities of daily living and the care of elderly patients with complex health conditions.

The size of the participating facilities and the nurse‐to‐patient ratio differed. For instance, in one facility, the nurse‐to‐patient ratio was approximately 1:5, and in another facility, the nurse‐to‐patient ratio was approximately 1:7. These differences were due to differences in institutional capacity, staffing and operational arrangements. All facilities were deemed to be suitable settings to study the association between the nursing work environment and resilience, as nurses in these institutions were directly involved with the clinical, emotional and organisational requirements of geriatric care.

The study was conducted in urban geriatric centres in Hanoi. Thus, the results might not be fully applicable to the geriatric nursing setting in rural areas, private institutions or less resource‐rich areas of Vietnam. This limitation in context has been noted in the limitations section.

### 3.3. Sample and Sampling

The study population comprised registered nurses working in geriatric care facilities in Hanoi. Eligible participants were registered nurses who were directly involved in patient care and had at least 6 months of continuous employment at their current facility. This criterion was applied to ensure that participants had sufficient exposure to the organisational work environment to provide informed and reliable evaluations of workplace conditions, consistent with approaches commonly adopted in studies examining nursing practice environments [22]. Nurses working exclusively in administrative roles, nursing students and those on extended leave during the data collection period were excluded.

This was a convenience study that involved all the qualified nurses in the chosen facilities who were invited to take part in the study. The power analysis was conducted through GPower, where the assumed level of effect size (*r* = 0.30), the level of significance (0.05) and the level of power (0.80) were used. The minimum sample size was determined as 84, and the sample finished with 222 nurses, which is more than the minimum in statistical power.

G + Power software was used to calculate a priori sample size to accomplish the desired statistical power in the correlational analysis. According to a medium effect size, the significance level of 0.05 and the desired power of 0.80, the minimum required sample size was calculated. A larger sample was aimed at addressing the potential nonresponse and incomplete information. The final sampled nurses constituting the final sample have been justified to have totalled 222 participants who met the inclusion criteria and gave full responses. This was larger than the minimum size of a sample, giving the results greater strength. A convenience sampling method was utilised, during which all eligible nurses in the sampled facilities were persuaded to take part. Out of the 240 nurses, 222 received the survey, resulting in a response rate of 92.5.

### 3.4. Instruments

A structured questionnaire, which included standardised tools to denote the nursing work environment and nurse resilience, was used to collect data that consisted of a demographic information section. Inclusion of instruments of results was only on those tools that were or will be directly analysed to ensure that such instruments are methodological and relevant.

#### 3.4.1. Practice Environment Scale of the Nursing Work Index (PES‐NWI) (Short Version)

The PES‐NWI short form consists of 10 items. This abbreviated instrument was developed by Gea‐Caballero et al. [23] based on Lake’s original framework to assess the nursing practice environment, defined as organisational factors that facilitate or hinder nurses’ ability to practise effectively and provide high‐quality care. The short form comprises three dimensions: (1) leadership and management of healthcare services; (2) nursing foundations for quality of care and relationships with other professionals, reflecting autonomy in decision‐making and self‐management of nursing practice and (3) adequacy of human resources. The instrument demonstrated good internal consistency reliability, with a Cronbach’s alpha coefficient of 0.816 for the overall scale and values exceeding 0.80 for all dimensions. The research tools employed in this study, such as the PES‐NWI and RAW scales, were translated into Vietnamese by a conventional back‐translation technique. To have linguistic and conceptual equivalence, a pilot test was conducted on 10 nurses. The final versions of the scales were tested on clarity, and scale scores were calculated by averaging the responses in each domain. Items, which were coded in reverse, were processed as per the standard procedures to prevent scoring errors.

#### 3.4.2. Resilience Assessment Tool

Nurse resilience was measured using the Resilience at Work (RAW) Scale, a validated 20‐item instrument developed by Winwood et al. [24]. This tool assesses resilience across five distinct dimensions: (1) living authentically, which encompasses knowing and adhering to personal values, deploying personal strengths, emotional awareness/regulation and maintaining physical well‐being; (2) finding your calling, associated with seeking work that provides a sense of purpose, belonging and alignment with core beliefs; (3) maintaining perspective, reflecting the capacity to reframe setbacks, manage negativity and maintain a solution‐focused mindset; (4) managing stress, which involves utilising work–life routines to manage everyday stressors and ensure relaxation and (5) building social networks, concerning the development of personal support systems and cooperative interactions within the workplace, including seeking and providing feedback and support.

Participants rated their agreement with each item on a Likert‐type scale, where higher scores indicated higher levels of workplace resilience. In this study, the instrument demonstrated robust reliability. Cronbach’s alpha coefficient for the total scale was 0.84, indicating strong internal consistency. Reliability values for the individual subscales were also satisfactory, ranging from 0.60 to 0.89, confirming the instrument’s suitability for assessing resilience among geriatric nurses.

Instruments were administered in Vietnamese following a rigorous translation and back‐translation process to ensure linguistic and conceptual equivalence. To determine the relevance and clarity, a pilot test was carried out with a limited number of nurses. Slight adjustments were made according to feedback in order to enhance understanding. Scale scores were calculated by averaging the item responses within each of the domains; higher scores were taken to indicate more preferable working conditions and resilience. Items with negative wording were coded, then reversed and analysed. Predefined Likert scale thresholds were used to interpret domain and overall scores.

### 3.5. Data Collection

The data were gathered through an online survey conducted from September to December 2022. The online method was selected to facilitate access to nurses working across various shifts and settings while minimising disruption to clinical workflows. Facility managers assisted in distributing the survey link to eligible nurses, accompanied by an information sheet that defined the study’s aim and participation criteria.

Participation was voluntary, and electronic informed consent was obtained prior to accessing the survey. A total of 240 nurses received the survey invitation, and 222 nurses completed all necessary sections, resulting in a high response rate. Incomplete responses were excluded from the analysis to ensure data integrity. This research followed the suggested reporting guidelines of online surveys and some components of the CHERRIES checklist [25]. The process was voluntary, and informed consent was taken electronically, and respondents could pull out at any time. To counteract the chances of having redundant responses, there were measures that were made to exclude any incomplete submission out of final analysis. Since data have been gathered as of 2022, the results can be viewed within the framework of the possible shifts in the healthcare system and the state of the workforce over the years. Data collection occurred between September and December 2022, and it is important to note that the dataset may not fully reflect recent changes in the healthcare system, particularly postpandemic.

Parts of the Checklist for Reporting Results of Internet E‐Surveys (Eysenbach, 2004) were taken into account when preparing the manuscript for the purposes of reporting the process of the online survey as transparently as possible. This change is due to better reporting of the finished data collection process and is not a post hoc methodological change. This reporting clarification was added in a postcollection manner, with data already collected.

### 3.6. Ethical Considerations

The study strictly adhered to ethical standards, and ethical approval was granted by the Institutional Ethics Review Committee (Reference No. TUA‐IERC‐015‐R02) before data collection commenced. Administrative permission was also secured from the management of the respective geriatric facilities. Participants were fully informed about the study’s purpose, procedures and their rights, including the right to withdraw at any time without penalty. Confidentiality and anonymity were strictly maintained throughout the study. No personally identifying data were recorded, and all information was stored in a secure location accessible only to authorised research team members. The research was conducted in accordance with internationally recognised ethical standards for research involving human participants.

### 3.7. Data Analysis

Data were analysed using the Statistical Package for the Social Sciences (SPSS) Version 26.0. To provide an overview of demographics, perception of the working environment and level of resilience, descriptive statistics were calculated. Appropriate measures of central tendency and dispersion were determined based on the distribution of the data.

Normality was assessed using both statistical tests and visual inspection prior to conducting inferential analysis. Pearson’s correlation analysis was employed to test the relationships between nurse resilience and nursing work environment dimensions. The statistical level of significance was set at *p* < 0.05. The findings from these analyses are presented in the following section.

## 4. Results

### 4.1. Participant Characteristics

A total of 222 nurses participated in the study. The mean age of the respondents was 30.33 years (SD = 7.98). The sample was predominantly female (77.0%), while male nurses accounted for the remaining 23.0%. Regarding professional qualifications, a significant proportion of the participants (69.3%) held a college or university degree. In terms of professional seniority, the workforce was relatively junior, with the majority (61.5%) possessing less than 5 years of work experience. Furthermore, the vast majority of the sample (81.1%) were primarily engaged in direct patient care roles.

### 4.2. Nursing Work Environment

The general attitudes towards the nursing working conditions were average among the sample, with some differences in the dimensions represented by the PES‐NWI.

#### 4.2.1. Participation in Management and Leadership

Table A1 presents the nurses’ perceptions of participation in management and leadership. The overall mean score was 3.28, corresponding to a verbal interpretation of ‘Strongly Agree’, indicating generally positive perceptions of the management environment.

Specifically, respondents rated opportunities for professional development most highly, with ‘Continuing education programs’ receiving the highest mean score (3.39), followed by ‘Active programmes for quality improvement’ (3.31). Both items fell within the ‘Strongly Agree’ range.

However, items related to direct leadership and decision‐making received comparatively lower scores. The perception that ‘The supervisor is a good manager and leader’ (3.22) and the opportunity to ‘Participate in policy decisions’ (3.18) fell within the ‘Agree’ category. This suggests that while nurses feel supported in terms of education and quality standards, their inclusion in decision‐making processes and their perception of leadership competence are areas with room for improvement.

#### 4.2.2. Focus on Nursing Care and Interdisciplinary Relationships

Table A2 presents the results regarding nursing foundations for quality of care and interdisciplinary relationships. The overall mean score was 3.22, interpreted as ‘Agree’, indicating a generally positive assessment of care quality and professional relationships.

Specifically, nurses rated the foundational aspects of care highly. The item ‘Common, well‐defined nursing philosophy’ received the highest score (3.41), followed by ‘Clinical competence of nurses’ (3.34) and ‘Nurse‐physician collaboration’ (3.26). All three items fell within the ‘Strongly Agree’ range, suggesting a robust professional culture and strong teamwork.

However, the structural aspect of care delivery received a comparatively lower rating. The item regarding the ‘Allocation of patients to promote continuity of care’ obtained the lowest mean score (2.89), falling into the ‘Agree’ category. This indicates that while the professional philosophy and collaboration are strong, the logistical assignment of patients remains a challenge to ensuring continuous care.

#### 4.2.3. Adequate Resources

Table A3 summarises the results regarding staffing and resource adequacy. The overall mean score was 3.02, interpreted as ‘Agree’. Notably, compared to other dimensions of the work environment (management and quality of care), this dimension received the lowest ratings, indicating it is a relative area of concern.

Specific analysis reveals that nurses perceive the quantity of staff to be slightly better than the quality of the staffing mix. The item ‘Sufficient employees to do the job’ had a mean of 3.09, whereas ‘Sufficient number of qualified nurses’ received the lowest score in the entire survey (2.96). This suggests that while the total headcount might be acceptable, there is a perceived shortage of highly qualified or specialised nurses for geriatric care.

In summary, nurses generally perceived their working environment as supportive, though significant variations existed across different dimensions. Dimensions related to leadership engagement and interdisciplinary interaction received higher ratings compared to staffing and resource adequacy. The consistently lower ratings assigned to staffing sufficiency highlight critical structural limitations within the studied geriatric care facilities. This suggests that while the professional culture and management support are relatively strong, the operational capacity, specifically regarding human resources, remains a challenge.

### 4.3. Nurse Resilience

This section presents the findings regarding the resilience levels of the nurse respondents. The assessment is based on five distinct dimensions: *Living Authentically and Healthily*, *Finding One’s Calling*, *Maintaining Perspective*, *Managing Stress* and *Building Social Networks*. Overall, the results indicate that nurses reported generally positive levels of resilience, particularly in areas related to healthy living practices, self‐reported stress management capacity and peer support. However, variability was observed across the dimensions, with comparatively lower scores in aspects related to boundary maintenance and professional feedback‐seeking. The detailed results for each dimension are presented in Tables A4–A8

Table A4 presents the descriptive statistics for the living authentically and healthily dimension of nurse resilience. The overall mean score was 3.35, indicating a generally positive level of resilience within this dimension.

Respondents reported the highest ratings for items related to healthy dietary practices and value alignment. Specifically, *‘I am careful about eating well and healthily’* yielded the highest mean score (*M* = 3.62), followed by *‘I have important core values that I hold fast into my work life’* (*M* = 3.51).

In contrast, items related to physical fitness and emotional regulation received comparatively lower mean scores, although they remained above the scale midpoint. *‘I have a good level of physical fitness’* recorded the lowest mean (*M* = 3.08), followed by *‘I can change my mood at work when I need to’* (*M* = 3.16), suggesting relative variability across aspects of this resilience dimension.

Table A5 presents the descriptive statistics for the finding one’s calling dimension of nurse resilience. The overall mean score was 3.10, corresponding to an ‘Agree’ level, indicating a generally positive but not strong endorsement of this dimension.

A distinction was observed between nurses’ perceived alignment with the profession and their perceived attachment to the workplace. The item *‘The work that I do fits well with my personal values and beliefs’* received the highest mean score (*M* = 3.29) and was the only item interpreted as ‘Strongly Agree’, suggesting a strong alignment between personal values and professional roles.

In contrast, items reflecting organisational attachment received comparatively lower ratings. Notably, *‘My workplace is somewhere where I feel that I belong’* recorded the lowest mean score (*M* = 2.86). This pattern suggests that while nurses perceive their work as meaningful and value‐consistent, their sense of belonging to the specific organisational setting is comparatively weaker.

Table A6 presents the descriptive statistics for the maintaining perspective dimension. The overall mean was 2.77, interpreted as ‘Agree’, reflecting a moderate capacity to maintain a balanced outlook amidst work challenges.

The results highlight a contrast between interpersonal resilience and work–life boundaries. On a positive note, respondents demonstrated strong resistance to social negativity and good emotional recovery. The highest mean score was recorded for ‘Nothing at work ever really ‘fazes me’ for long’ (*M* = 3.06), indicating an ability to bounce back from stressors. Furthermore, participants disagreed with the statement ‘Negative people at work tend to pull me down’ (*M* = 2.49), suggesting that they are capable of shielding themselves from the negative emotions of colleagues.

However, compartmentalising work stress proved more difficult. Respondents agreed that ‘When things go wrong at work, it usually tends to overshadow the other parts of my life’ (*M* = 2.77). This implies that while nurses are resilient against immediate workplace negativity, they struggle to prevent professional setbacks from intruding into their personal lives.

Table A7 presents the descriptive statistics for the managing stress dimension of nurse resilience. The overall mean score was 3.38, interpreted as ‘Strongly Agree’. This indicates that the nurses generally possess effective strategies for handling workplace pressure.

Respondents expressed the highest confidence in their coping mechanisms. The item ‘I have developed some reliable ways to relax when I am under pressure at work’ received the highest mean score (*M* = 3.66), followed closely by ‘I have developed some reliable ways to deal with the stress of challenging events’ (*M* = 3.41).

However, work–life boundaries appeared to be a vulnerable area. The item ‘I am careful to ensure my work does not dominate my personal life’ received the lowest rating (*M* = 3.05) and was the only item falling into the ‘Agree’ category. This suggests that while nurses are skilled at managing stress *within* the workplace, they face challenges in preventing work demands from encroaching on their personal lives.

Table A8 summarises the descriptive statistics for the Building Social Network dimension of nurse resilience. The findings indicate a high level of perceived social support among respondents, with an overall mean score of 3.28, interpreted as *Strongly Agree*.

Nurses reported particularly strong confidence in their workplace emotional support systems. The item *‘I have friends at work whom I can rely on to support me when I need it’* received the highest mean score (*M* = 3.57), followed by *‘I have a strong and reliable network of supportive colleagues at work’* (*M* = 3.35).

However, a distinction emerged between interpersonal support and feedback‐seeking behaviours. The item *I often ask for feedback so that I can improve my work performance’* recorded the lowest mean score (*M* = 2.88), falling within the *Agree* category. This suggests that while nurses perceive strong mutual support and collegial trust, they may be comparatively less inclined to actively seek evaluative feedback for professional development.

Collectively, these findings reveal a distinct resilience profile among geriatric nurses. Respondents demonstrated strong internal and social resources, reflected in a high alignment with personal values, a solid network of supportive colleagues and a generally positive self‐reported capacity to manage work‐related stress.

However, the data also highlight critical areas of vulnerability, particularly in boundary maintenance and professional openness. The comparatively lower scores in *Maintaining Perspective*, together with difficulties preventing work‐related stress from intruding into personal life (as reflected in the *Managing Stress* dimension), suggest challenges in effectively compartmentalising professional and personal roles. Furthermore, although nurses reported strong reliance on peer support, the relatively lower tendency to seek feedback (within the *Building Social Network* dimension) may indicate a hesitancy to engage in evaluative professional dialogue, potentially limiting opportunities for reflective learning and growth.

### 4.4. Relationship Between Work Environment and Resilience

Table A9 presents the correlational analysis between the aggregate scores of the self‐assessed work environment and nurse resilience among geriatric nurses. The results indicate a statistically significant moderate positive correlation between the overall work environment and resilience (*r* = 0.534, *p* = 0.001). This finding confirms the primary hypothesis that generally, more favourable perceptions of the work environment are associated with higher overall levels of nurse resilience.

To gain a deeper understanding of this relationship, the study further examined the associations between specific dimensions of the work environment and components of nurse resilience, as summarised in Table A10, revealing relationships that vary significantly in both magnitude and direction (*p* < 0.05).

#### 4.4.1. Impact on Professional Adaptation

Among the resilience dimensions, managing stress showed a strong positive correlation with focus on nursing care and interdisciplinary relationships (*r* = 0.803). This indicates that an environment emphasising high‐quality care and teamwork significantly enhances the nurses’ professional capability to cope with workplace pressure. Similarly, all environmental dimensions were positively correlated with finding one’s calling, although the strength of these associations was generally low, reflecting a modest alignment between workplace conditions and the nurses’ sense of professional purpose.

#### 4.4.2. Impact on Personal Boundaries (The Trade‐Off)

In contrast, significant inverse relationships were observed between focus on nursing care and two personal resilience dimensions: maintaining perspective (*r* = −0.730) and living authentically (*r* = −0.703). These findings imply a potential trade‐off: while a high emphasis on clinical care drives professional coping strategies, it appears to come at the cost of personal work–life boundaries, making it harder for nurses to maintain a balanced outlook or live authentically outside of their professional role.

*The Role of Resources*: Adequate resources demonstrated a distinct pattern. It was strongly positively correlated with maintaining perspective (*r* = 0.746) and moderately with living authentically (*r* = 0.624), suggesting that structural support is critical for maintaining personal balance. Conversely, resource adequacy showed a significant negative correlation with managing stress (*r* = −0.583), indicating differing dynamics where nurses in well‐resourced settings may rely less on active stress‐coping mechanisms.

#### 4.4.3. Social Network Dynamics

Additionally, the dimension building social network exhibited significant negative correlations with all work environment dimensions. This inverse association suggests a potential resource trade‐off rather than a simple lack of need for support. In geriatric settings where the organisational focus on nursing care quality and leadership adherence is intense, nurses may channel their limited time and emotional energy primarily into formal professional interactions and patient care. Consequently, this intense professional immersion may inadvertently crowd out the opportunities or cognitive capacity required to cultivate and maintain broader social networks, reinforcing the trade‐off between professional dedication and personal psychosocial expansion observed in other dimensions.

Overall, while the findings confirm that the work environment is significantly associated with nurse resilience, the relationship is multidimensional. Structural and professional support enhances specific adaptive capacities (like stress management) but may simultaneously impose challenges on personal boundaries, highlighting the nonuniform nature of resilience in the geriatric care context.

The GPower software was used to calculate the sample size, taking the following as the inputs of a bivariate correlation analysis: medium effect size (*r* = 0.30), significance level (−) = 0.05, statistical power = 0.80 and two tails. A minimum sample size of 84 individuals was required, so the sample size of 222 met the requirement and increased the strength of the results.

## 5. Discussion

### 5.1. Summary of Key Findings

Findings reveal that nurses perceive their work environment as generally supportive, particularly in leadership and care quality, despite notable resource constraints. Resilience profiles were heterogeneous; nurses excelled in stress management but struggled with boundary maintenance. Significantly, while the overall work environment positively correlates with resilience (*r* = 0.534), dimension‐specific analysis highlights a trade‐off: a strong focus on clinical care reinforces professional coping mechanisms yet appears to compromise personal work–life perspective.

### 5.2. Work Environment and Nurse Resilience

The positive association observed between the nursing work environment and nurse resilience in this study is consistent with international literature identifying organisational context as a key correlate of nurses’ psychological well‐being. Evidence from high‐income countries has repeatedly shown that supportive leadership, adequate staffing and collaborative working conditions are associated with higher resilience, lower burnout and improved nurse retention. The present findings extend this body of evidence to geriatric care settings in Vietnam, suggesting that the relevance of organisational support to nurse resilience persists across different healthcare systems and levels of resource availability.

Within the Asian nursing literature, research examining the relationship between work environment and psychological outcomes has increased in recent years; however, most studies have focused on acute care hospital contexts. By contrast, the current study addresses an important gap by examining geriatric facilities, a setting characterised by sustained emotional labour and long‐term care demands. The findings indicate that organisational dimensions—particularly leadership participation, interdisciplinary relationships and resource adequacy—are meaningfully associated with multiple aspects of nurse resilience in this context. This supports previous observations that organisational support structures play an important role in sustaining adaptive functioning among nurses working in emotionally and physically demanding environments [26].

Importantly, the results also point to the multidimensional and nuanced nature of resilience in geriatric nursing. While a strong focus on nursing care and interdisciplinary collaboration was positively associated with stress management capacities, it was inversely associated with dimensions related to maintaining perspective and living authentically. Given the correlational design of the study, these findings should be interpreted cautiously; nevertheless, they suggest that intensive professional engagement may coexist with challenges in maintaining personal boundaries and work–life balance. Similar patterns have been discussed in the literature, where high professional commitment in caregiving roles is linked to both adaptive coping and increased vulnerability to role spillover [26].

In the Vietnamese context, these patterns may reflect broader structural constraints within a developing healthcare system, including staffing shortages, limited resources and comparatively lower institutional investment in geriatric care [22]. The observed associations suggest that even incremental improvements in the work environment may be linked to meaningful enhancements in nurse resilience. However, the findings also underscore the importance of organisational‐level interventions that address structural conditions, rather than relying solely on individual resilience, to support sustainable well‐being among geriatric nurses.

### 5.3. Leadership, Staffing and Interdisciplinary Collaboration

From a nursing management perspective, the findings identify leadership, staffing and interdisciplinary collaboration as pivotal leverage points for enhancing nurse resilience. The positive association between leadership participation and resilience suggests that inclusive and supportive leadership practices can significantly bolster nurses’ capacity to handle workplace stressors [8]. However, given the study’s finding that leadership involvement also showed relationships with personal boundary dimensions, it is crucial for managers to support professional autonomy without micromanaging, thereby promoting a sense of control and professional identity that respects the nurses’ need for authenticity.

The issue of staffing and resource adequacy emerged as particularly relevant, given the comparatively lower scores recorded in this dimension. These findings have substantial implications for nurse managers regarding resource distribution. As indicated by the data, adequate resources are strongly linked to the nurses’ ability to maintain perspective and work–life balance. Consequently, chronic understaffing not only increases workload but also erodes the structural support necessary for nurses to mentally detach from work and recharge, thereby compromising long‐term resilience.

Furthermore, interdisciplinary collaboration was identified as a critical factor, particularly in its strong correlation with stress management capacities. In geriatric care, where patient needs are complex, teamwork and effective communication are essential [7]. The findings suggest that role strain can be mitigated through robust interaction with physicians, therapists and support staff, which enhances care coordination and provides emotional buffering. Therefore, nursing managers should prioritise cultivating collaborative cultures by dismantling rigid hierarchies and fostering open communication, ensuring that teamwork serves as a protective resource rather than an additional source of conflict.

### 5.4. Cultural and Contextual Interpretation Within Vietnam

The results of this study must be interpreted within the specific cultural and organisational context of Vietnam. Vietnamese healthcare organisations are often characterised by hierarchical structures and centralised decision‐making, reflecting broader cultural norms that value authority and seniority. These characteristics can inadvertently restrict the involvement of nurses in organisational activities. Although respect for authority is culturally embedded, rigid structures may limit opportunities for shared governance and the exercise of professional autonomy unless complemented by inclusive leadership practices. These dynamics likely contribute to the moderate scores observed in this study regarding participation in management and leadership, suggesting that while nurses perceive a degree of organisational support, they may not yet feel fully empowered as active partners in decision‐making.

The nursing work environment is further shaped by systemic resource constraints. Vietnam is experiencing one of the fastest rates of population ageing globally, placing increasing pressure on geriatric services that often receive fewer investments compared to acute care sectors. At a global level, the *State of the World’s Nursing 2020* report highlights widespread nursing workforce shortages and underscores the need for strategic investments in nursing education, employment and leadership development to strengthen health systems [1]. These global challenges resonate with the Vietnamese context, where workforce limitations and uneven resource allocation may intensify the demands placed on geriatric nurses. Empirical evidence also points to gaps in geriatric‐specific knowledge and preparedness among healthcare providers [18], as well as concerns regarding the willingness and readiness of younger nurses to engage in elder care [19]. The migration of experienced nurses to other sectors or international labour markets further exacerbates staffing pressures, reinforcing perceptions of resource insufficiency and increasing the workload of remaining staff.

Despite these structural constraints, the positive associations identified in this study indicate that organisational support and leadership practices remain critical determinants of nurse resilience. Consistent with WHO [1] recommendations, strengthening nursing leadership and improving practice environments can enhance nurses’ capacity to function effectively even in resource‐limited settings. In the Vietnamese context, effective leadership may not require dismantling hierarchical norms but rather adapting them to foster open communication, recognition and meaningful involvement of nurses. Accordingly, interventions aimed at enhancing resilience should be culturally sensitive, balancing respect for organisational structures with genuine professional and emotional support for geriatric nurses.

### 5.5. Theoretical Implications

The results of this research validate and reinforce the applicability of the Neuman systems model in nursing management studies. The positive correlation found between work environment features and resilience aligns with the model’s conceptualisation of individuals interacting with environmental stressors. In this framework, supportive organisational conditions—such as adequate resources and inclusive leadership—function as a reinforcement of the nurses’ flexible lines of defence. By strengthening this defensive buffer, the organisation helps mitigate the impact of workplace stressors, thereby preventing system instability and facilitating the development of adaptive responses [20].

Furthermore, these findings contribute to nursing resilience theory by framing resilience as a dynamic, context‐dependent process rather than a static individual trait. The study provides empirical evidence that resilience is significantly shaped by external factors, including leadership participation, resource adequacy and professional relationships. This theoretical alignment challenges traditional views that situate the responsibility for coping solely on the individual nurse. Instead, it underscores the critical role of the organisation, suggesting that resilience‐building interventions must target structural and systemic levels to be truly effective.

## 6. Implications for Nursing Management

The results of this research have major implications for the management of nursing in geriatric care facilities.

First, leadership development must be prioritised, with an emphasis on relational and participatory leadership styles. Nursing managers who actively engage with staff, promote shared decision‐making and provide constructive feedback foster environments where resilience can thrive. Training programmes should focus on essential competencies such as communication skills, emotional intelligence and strategies to address staff well‐being [1].

Second, it is crucial to enhance nurse involvement in organisational decisions. The sense of ownership and professional agency is significantly improved through shared governance models and frequent opportunities for nurses to participate in policy and practice decisions [8]. This participation builds resilience by ensuring that nurses feel their voices are heard and valued within the organisation.

Third, strategic planning for staffing and resource allocation is essential. Nursing managers should advocate for staffing models that reflect the complexity of geriatric care rather than just patient numbers. While systemic constraints may exist, transparent communication regarding resource allocation and efforts to optimise skill mix can help mitigate the adverse effects of resource limitations on nurse resilience [22].

Fourth, resilience‐building interventions should be integrated into organisational practice rather than implemented as standalone, temporary programmes. Leadership and structural supports should be supplemented with mentorship initiatives, peer support systems and reflective practice sessions. Importantly, these strategies must be reinforced by organisational policies acknowledging that resilience is a systemic responsibility, not solely an individual task [12, 26].

## 7. Limitations

This study acknowledges several limitations that should be considered when interpreting the results.

First, the cross‐sectional design limits the ability to establish causal relationships between the nursing work environment and resilience. As resilience is a dynamic process that may fluctuate over time or in response to changing organisational conditions, a longitudinal approach would be required to assess these temporal changes and causal directions [21].

Second, the reliance on self‐reported measures introduces the potential for response bias. Participants’ responses may be influenced by social desirability or their transient emotional states at the time of data collection, rather than reflecting their objective reality.

Third, the study was conducted exclusively in geriatric units within Hanoi, which may limit the generalisability of the findings to other regions of Vietnam or different healthcare settings. Organisational cultures and resource availability can vary significantly between urban and rural areas, or between public and private sectors, potentially affecting both work environment perceptions and resilience levels.

Finally, although validated instruments were used, cultural nuances may affect interpretation. While the scales demonstrated acceptable reliability, the translation and cultural adaptation of Western‐developed instruments (such as the resilience scale) into the Vietnamese context may still carry subtle variations in how specific items are perceived and understood by local respondents. Consequently, future research utilising mixed‐methods approaches and expanding to diverse geographical settings is recommended to validate and extend these findings. The use of online self‐administered surveys may have introduced selection bias as the survey was distributed via institutional channels, potentially limiting the diversity of responses.

## 8. Conclusion

This study provides empirical evidence that the nursing work environment is significantly associated with the resilience of nurses working in geriatric centres in Hanoi, Vietnam. While the overall relationship is positive, the findings reveal a nuanced dynamic: supportive work environments were associated with greater resilience, yet high clinical demands may inadvertently strain nurses’ personal work–life boundaries. This highlights that resilience is not merely an individual trait but a context‐dependent process deeply influenced by organisational conditions.

For nursing management, these results underscore the urgent need for a paradigm shift—moving away from viewing resilience solely as an individual responsibility towards recognising it as an organisational outcome. To build a strong and sustainable geriatric workforce, nursing managers must prioritise culturally sensitive leadership that balances structural authority with emotional support, while actively addressing chronic resource and staffing constraints. Ultimately, strengthening the nursing work environment is not just a strategy for staff retention; it is a fundamental prerequisite for ensuring high‐quality care for the elderly population in Vietnam.

Furthermore, an online self‐administered survey could have resulted in selection bias because the questionnaire could have been distributed via institutional channels, and therefore, people could have joined in based on accessibility, availability or personal interest. Furthermore, the study was carried out in selected urban geriatric facilities in Hanoi that were sampled by convenience sampling; so the results should be cautiously applied to the rural geriatric facilities, private facilities and other places in Vietnam. The institutional capacity, nurse‐to‐patient ratio, staffing composition and geriatric resources are factors that may affect the nurse’s perception of the work environment as well as the nurse’s resilience. Further studies with more facilities in urban and rural areas are suggested to improve the generalisability of the results. As the study used a cross‐sectional correlational design, the results of the study are not causal but rather associations.

## Author Contributions

Thuy Thi Ngo: conceptualisation, methodology, data collection, data analysis and writing–original draft preparation.

Lydia T. Manahan: data collection, formal analysis and writing–review and editing.

Edreck D. Estioko: formal analysis and writing–review and editing.

Huong Thi Thu Pham: writing–review and editing.

## Funding

This research was not funded by any external agencies. No financial support was received for this study.

## Ethics Statement

The study was conducted following ethical guidelines outlined in the Declaration of Helsinki. Ethical approval was obtained from the Institutional Ethics Review Committee of Phenikaa University (Reference No. TUA‐IERC‐015‐R02).

All participants were fully informed about the purpose of the study, the voluntary nature of their participation and their right to withdraw at any point without any penalty. Informed consent was obtained electronically before participation.

The confidentiality and anonymity of participants were ensured, with no personally identifiable information recorded in the study.

## Conflicts of Interest

The authors declare no conflicts of interest.

## Data Availability

The data that support the findings of this study are available from the corresponding author, Dr. Thuy Thi Ngo, upon reasonable request.

## Appendix A

**TABLE A1:** Nurses’ perceptions of participation in management and leadership.

Item	Mean	SD	Interpretation
Continuing education programmes are available	3.39	NR	Strongly agree
Active programme for quality improvement are present	3.31	NR	Strongly agree
The supervisor is a good manager and leader	3.22	NR	Agree
Nurses participate in policy decisions	3.18	NR	Agree
**Overall mean**	**3.28**	**NR**	**Strongly Agree**

*Note:* Bold values denote overall domain scores.

**TABLE A2:** Nursing foundations for quality of care and interdisciplinary relationships.

Item	Mean	SD	Interpretation
Common, well‐defined nursing philosophy	3.41	NR	Strongly agree
Clinical competence of nurses	3.34	NR	strongly agree
Nurse–physician collaboration	3.26	NR	Strongly agree
Allocation of patients promotes continuity of care	2.89	NR	Agree
**Overall mean**	**3.22**	**NR**	**Agree**

*Note:* Bold values denote overall domain scores.

**TABLE A3:** Staffing and resource adequacy.

Item	Mean	SD	Interpretation
Sufficient employees to do the job	3.09	NR	Agree
Sufficient number of qualified nurses	2.96	NR	Agree
**Overall mean**	**3.02**	**NR**	**Agree**

*Note:* Bold values denote overall domain scores.

**TABLE A4:** Living authentically and healthily.

Item	Mean	SD	Interpretation
I am careful about eating well and healthily	3.62	NR	Strongly agree
I have important core values that I hold fast in my work life	3.51	NR	Strongly agree
I can change my mood at work when I need to	3.16	NR	Agree
I have a good level of physical fitness	3.08	NR	Agree
**Overall mean**	**3.35**	**NR**	**Positive resilience level**

*Note:* Bold values denote overall domain scores.

**TABLE A5:** Finding One’s calling.

Item	Mean	SD	Interpretation
The work that I do fits well with my personal values and beliefs	3.29	NR	Strongly agree
My workplace is somewhere where I feel that I belong	2.86	NR	Agree
**Overall mean**	**3.10**	**NR**	**Agree**

*Note:* Bold values denote overall domain scores.

**TABLE A6:** Maintaining perspective.

Item	Mean	SD	Interpretation
Nothing at work ever really fazes me for long	3.06	NR	Agree
When things go wrong at work, it usually tends to overshadow the other parts of my life	2.77	NR	Agree
Negative people at work tend to pull me down	2.49	NR	Disagree/low agreement
**Overall mean**	**2.77**	**NR**	**Agree**

*Note:* Bold values denote overall domain scores.

**TABLE A7:** Managing stress.

Item	Mean	SD	Interpretation
I have developed some reliable ways to relax when I am under pressure at work	3.66	NR	Strongly agree
I have developed some reliable ways to deal with the stress of challenging events	3.41	NR	Strongly agree
I am careful to ensure my work does not dominate my personal life	3.05	NR	Agree
**Overall mean**	**3.38**	**NR**	**Strongly agree**

*Note:* Bold values denote overall domain scores.

**TABLE A8:** Building social networks.

Item	Mean	SD	Interpretation
I have friends at work whom I can rely on to support me when I need it	3.57	NR	Strongly agree
I have a strong and reliable network of supportive colleagues at work	3.35	NR	Strongly agree
I often ask for feedback so that I can improve my work performance	2.88	NR	Agree
**Overall mean**	**3.28**	**NR**	**Strongly agree**

*Note:* Bold values denote overall domain scores.

**TABLE A9:** Correlation between overall nursing work environment and nurse resilience.

Variables	*r*	*p* value	Interpretation
Overall nursing work environment and overall nurse resilience	0.534	0.001	Moderate positive significant correlation

**TABLE A10:** Correlation between work environment dimensions and nurse resilience domains.

Work environment dimension	Resilience domain	*r*	*p*‐value	Interpretation
Focus on nursing care and interdisciplinary relationships	Managing stress	0.803	< 0.05	Strong, positive, significant correlation
Focus on nursing care and interdisciplinary relationships	Maintaining perspective	−0.730	< 0.05	Strong, negative, significant correlation
Focus on nursing care and interdisciplinary relationships	Living authentically	−0.703	< 0.05	Strong, negative, significant correlation
Adequate resources	Maintaining perspective	0.746	< 0.05	Strong, positive, significant correlation
Adequate resources	Living authentically	0.624	< 0.05	Moderate, positive, significant correlation
Adequate resources	Managing stress	−0.583	< 0.05	Moderate, negative, significant correlation
Work environment dimensions	Building social networks	Negative correlations reported	< 0.05	Significant inverse associations
